# Early rise in brain damage markers and high ICOS expression in CD4+ and CD8+ T cells during checkpoint inhibitor-induced encephalomyelitis

**DOI:** 10.1136/jitc-2021-002732

**Published:** 2021-07-02

**Authors:** Sara Bjursten, Ankur Pandita, Zhiyuan Zhao, Charlotta Fröjd, Lars Ny, Christer Jensen, Tobias Ullerstam, Henrik Jespersen, Jan Borén, Malin Levin, Henrik Zetterberg, Anna Rudin, Max Levin

**Affiliations:** 1 Department of Oncology, Institute of Clinical Sciences, University of Gothenburg Sahlgrenska Academy, Goteborg, Sweden; 2 Department of Oncology, Sahlgrenska University Hospital, Goteborg, Sweden; 3 Department of Molecular and Clinical Medicine/Wallenberg Laboratory, Institute of Medicine, University of Gothenburg Sahlgrenska Academy, Goteborg, Sweden; 4 Department of Neuroradiology, Sahlgrenska University Hospital, Goteborg, Sweden; 5 Department of Anesthesiology and Intensive Care, Sahlgrenska University Hospital, Goteborg, Sweden; 6 Department of Oncology, Akershus University Hospital, Lorenskog, Norway; 7 Department of Psychiatry and Neurochemistry, University of Gothenburg Institute of Neuroscience and Physiology, Goteborg, Sweden; 8 Clinical Neurochemistry Laboratory, Sahlgrenska University Hospital, Goteborg, Sweden; 9 Department of Neurodegenerative Disease, UCL Queen Square Institute of Neurology, London, UK; 10 UK Dementia Research Institute, UCL, London, UK; 11 Department of Rheumatology and Inflammation Research, Institute of Medicine, University of Gothenburg Sahlgrenska Academy, Goteborg, Sweden

**Keywords:** immunotherapy, melanoma, autoimmunity, CD4-Positive T-Lymphocytes, CD8-Positive T-Lymphocytes

## Abstract

We report a case of rapid eradication of melanoma brain metastases and simultaneous near-fatal encephalomyelitis following double immune checkpoint blockade. Brain damage marker S-100B and C reactive protein increased before symptoms or signs of encephalomyelitis and peaked when the patient fell into a coma. At that point, additional brain damage markers and peripheral T cell phenotype was analyzed. The analyses were repeated four times during the patient’s recovery. Axonal damage marker neurofilament light polypeptide (NFL) and astrocytic damage marker glial fibrillar acidic protein (GFAP) were very high in blood and cerebrospinal fluid and gradually normalized after immunosuppression and intensive care. The costimulatory receptor inducible T cell costimulatory receptor (ICOS) was expressed on a high proportion of CD4+ and CD8+T cells as encephalomyelitis symptoms peaked and then gradually decreased in parallel with clinical improvement. Both single and double immune checkpoint inhibitor-treated melanoma patients with other serious immune-related adverse events (irAE) (n=9) also expressed ICOS on a significantly higher proportion of CD4+ and CD8+T cells compared with controls without irAE (n=12). In conclusion, our results suggest a potential role for ICOS on CD4+ and CD8+T cells in mediating encephalomyelitis and other serious irAE. In addition, brain damage markers in blood could facilitate early diagnosis of encephalitis.

Immune checkpoint blockade increases survival in patients with metastatic malignant melanoma, renal cell carcinoma, and lung cancer.^1–4^ Double immune checkpoint blockade activates T cells by blocking the inhibitory receptors programmed cell death 1 (PD-1) and cytotoxic T-lymphocyte-associated protein 4 (CTLA-4) simultaneously, thereby promoting T cells to kill cancer cells. However, it increases the risk of autoimmune reactions in healthy tissue, immune-related adverse events (irAE).^5^ Any organ system can be affected by irAE and among the most feared is engagement of the brain, encephalitis.^6–8^ With the growing use of immune checkpoint blockade in cancer patients, encephalitis cases are expected to increase. A clinical challenge is to quickly and accurately diagnose encephalitis, which is difficult to distinguish from conditions such as progression of brain metastasis or infection that give rise similar symptoms. Also, little is known about the T cell mediated mechanisms promoting encephalitis and other serious irAE.

## Encephalomyelitis case and controls

### Encephalomyelitis case

**Figure 1:**
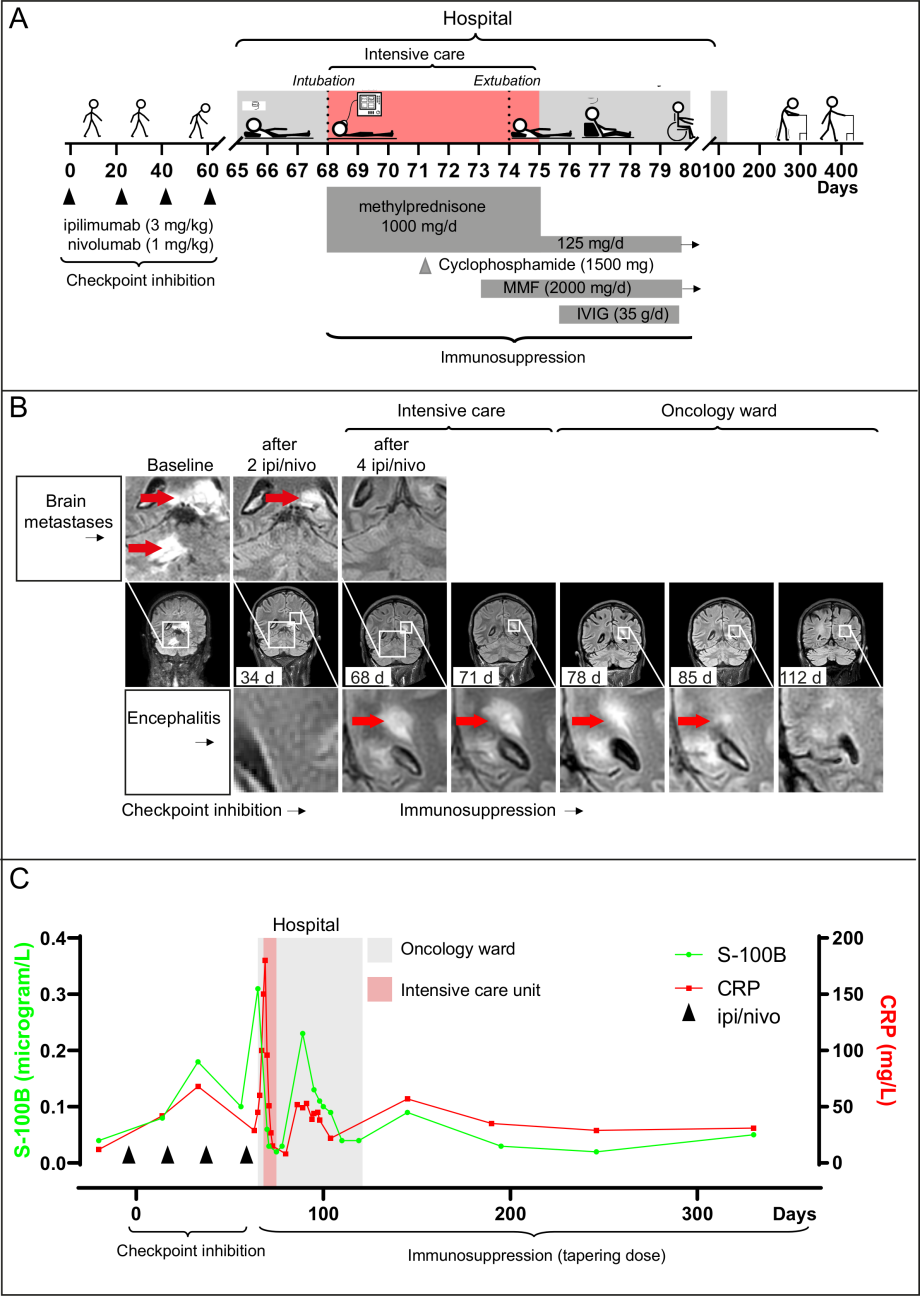
Regression of encephalomyelitis induced by double immune checkpoint blockade. (A) shows a clinical overview. (B) shows regression of brain metastases and progression of encephalitis on MRI scans. At baseline, the patient had a 25 mm metastasis in the left portion of the splenium corpus callosum and an 18 mm metastasis in the right cerebellar hemisphere (red arrows). After two treatments (34 days), both metastases had partially regressed. After four treatments (68 days), regression was complete but new diffuse lesions were seen in the posterior horns of the lateral ventricles indicating encephalitis (red arrows). MRI scans were unchanged at day 71 and showed gradual decrease of the lesions in the brain at 78 and 85 days and complete resolution at 112 days. (C) shows serum levels of S-100B and C reactive protein (CRP), starting at baseline. The highest measurements coincided with the most severe symptoms of encephalitis. However, the first peak in S-100B and CRP levels occurred before the patient had any symptoms or radiological findings of encephalitis, suggesting a potential biomarker for early detection. IPI, ipilimumab; IVIG, intravenous immunoglobulin; MMF, mycophenolate mofetil; nivo, nivolumab.

A 67-year-old man with metastases of melanoma in the brain, adrenal glands, lung, subcutis, and lymph nodes started double immune checkpoint blockade; PD1-inhibitor nivolumab (1 mg/kg) and the CTLA-4 inhibitor ipilimumab (3 mg/kg) given four times at 3-week intervals (figure 1A). After two treatments, MRI scans (MRI) showed regression of the brain metastases (figure 1B). The day after the fourth treatment, he developed a fever (39.1°C), elevation of C reactive protein (CRP) (45 mg/L), and dizziness. He was admitted to the oncology ward at Sahlgrenska University Hospital and was given IV antibiotics. After 2 days, he developed paraparesis in his lower limbs, decreased consciousness, and respiratory failure. His CRP level increased to 130 mg/L.

He was admitted to the intensive care unit (ICU), intubated, and put on mechanical ventilation. On ICU day 1, MRI showed complete regression of his brain metastases but new diffuse lesions in the brain, brainstem, and cerebellum with a bilateral and asymmetric pattern (figure 1B), indicating acute disseminated encephalomyelitis.^9^ Analyses of cerebrospinal fluid (CSF) suggested autoimmune encephalitis (online supplemental table S1). Immunosuppression with methylprednisolone (1 g daily intravenously) was started. The next day he was alert during sedation stops but required respiratory support, reflecting brain stem damage. MRI on ICU day 3 revealed unchanged brain lesions and lesions in the spinal cord. Cyclophosphamide was administered (1.5 g intravenously as a single dose), but his condition did not improve. On ICU day 6, mycophenolate mofetil (1 g two times per day intravenously) was started to inhibit T and B cell proliferation.^10^ The next day, he had improved and could be extubated and transferred back to the oncology ward. He had flaccid paraparesis in his lower limbs with minor residual sensation and urine and fecal incontinence. High-dose cortisone and mycophenolate mofetil were continued, and intravenous immunoglobulins were given (35 g daily for 5 days). MRI 10 and 17 days after ICU admission revealed gradual decrease in lesions in the brain and spinal cord (figure 1B).

10.1136/jitc-2021-002732.supp1 Supplementary data

After 6 weeks, the patient was discharged from hospital care. Now, he had regained most sensory functions, and he could sit up and operate a wheelchair. After 10 weeks, the inflammatory lesions had resolved on MRI. The immunosuppressant treatment was tapered and permanently discontinued after 14 months. More than 2 years after the first dose of double immune checkpoint blockade, the patient no longer suffers from fecal incontinence and he can walk more than 100 steps with assistive devices. By becoming more independent, the patient’s quality of life has improved significantly and he remains tumor-free.

### Control patients with or without irAE

In addition to the encephalomyelitis patient, we also analyzed T cell characteristics in checkpoint inhibitor-treated patients with (n=9) or without other irAE (n=12) (table 1). The irAE were moderate to severe and occurred during double (n=2, including the encephalomyelitis patient) or single (PD-1) inhibition (n=8). Similarly, samples were obtained from patients without irAE during double (n=2) or single (PD-1) checkpoint inhibition (n=10). In addition, we performed repeated analysis of cytokines and soluble checkpoint proteins in CSF from the encephalomyelitis patient. As controls for this analysis, we used CSF from patients with autoimmune systemic lupus erythematosus without encephalitis (n=4).

**Table 1:** Patient characteristics

Age	Sex	BRAF	Tumor stage*	Treatment	Time to irAE/test†	Affected organ(s)	Grade (CTCAE 5)‡	Treatment of irAE
68	M	V600K	IV	Ipi+Nivo	2 months	Brain and spinal cord§	4	CORT, MMF, IVIG, CP
52	F	Wt	IV	Ipi+Nivo	2 months	Liver	4	CORT, MMF
72	F	Not known	IV	Pembro	3 months	Blood (neutropenia)	4	CORT, filgrastim
72	M	V600	IV	Pembro	13 months	Colon	3	CORT, infliximab
55	F	V600	IV	Nivo	14 months	Joints and muscles	3	CORT, MTX
38	F	Wt	IV	Nivo¶	5 months	Joints and muscles	3	CORT
83	F	V600E	IV	Nivo	1 month	Colon	3	CORT, infliximab
53	M	V600K	IV	Nivo	11 months	Lungs	2	CORT
80	F	Wt	IV	Nivo	10 months	Lungs	2	CORT
74	M	V600K	IV	Nivo±Relatlimab	2 months	Joints and muscles	2	CORT
								
71	M	Wt	IV	Ipi+Nivo	9 months	No irAE	0	none
80	M	Wt	IV	Ipi+Nivo	2 months	No irAE	0	none
80	M	V600K	IV	Pembro	5 months	No irAE	0	none
77	F	Wt	IV	Pembro	4 months	No irAE	0	none
82	F	Wt	IV	Pembro	3 months	No irAE	0	none
76	M	V600E	IV	Pembro	3 months	No irAE	0	none
67	M	V600	IV	Pembro	2 months	No irAE	0	none
68	M	V600E	IV	Nivo	4 months	No irAE	0	none
62	F	V600E	IV	Nivo	3 months	No irAE	0	none
57	M	V600E	IV	Nivo	2 months	No irAE	0	none
68	M	Wt	III	Nivo	1 month	No irAE	0	none
93	M	Wt	III	Nivo	1 month	No irAE	0	none

*Stage III: locally advanced disease. Stage IV: metastatic disease.

†Time from treatment start until adverse event (and sample) or time from treatment start until sample in controls without irAE.

‡CTCAE are a set of criteria for the standardized classification of adverse effects of cancer drugs. The scale ranges from grade 0 to grade 5. Grade 0 is no adverse event. Grade one adverse events have no or mild symptoms with or without laboratory abnormalities whereas grade five events are lethal.

§Encephalomyelitis patient.

¶irAE during Nivolumab monotherapy. Ipi/Nivo treatment previously without any side effects.

CORT, corticosteroids; CP, cyclophosphamide; CTCAE, Common Terminology Criteria for Adverse Events; Ipi, ipilimumab; irAE, immune related adverse event; IVIG, intravenous immunoglobuline; MMF, mycophenolate mofetil; MTX, methotrexate; Nivo, nivolumab; Pembro, pembrolizumab; wt, wild type.

## Methods

T cell characteristics, brain damage markers, and soluble checkpoint proteins were analyzed as described in online supplemental appendix.

## Results

### Incidence of encephalitis

The number of reported cases of encephalitis following both single and double immune checkpoint blockade is increasing and the fatality rate is high (WHO global database VigiBase, online supplemental figure S2).

### Brain damage markers and inflammation

Retrospective analysis of blood tests unexpectedly revealed a distinct pattern that was not evident during the patient’s stay. The brain damage marker S-100B and the inflammatory marker CRP covaried strikingly over time (figure 1C), and levels of both were elevated after two treatments with ipilimumab/nivolumab. At that point, the patient had no symptoms, and MRI showed regression of the brain metastases and no signs of encephalitis. After the fourth and final treatment, S-100B and CRP peaked, indicating combined inflammation and brain damage. The patient rapidly deteriorated, and MRI showed acute disseminated encephalomyelitis. These results suggest that brain damage markers in blood may indicate encephalitis before the appearance of typical signs on MRI or clinical neurological symptoms.

Extensive analysis of blood and CSF confirmed brain damage and inflammation (online supplemental figure S3, tables S1 and S4) but showed no signs of infection or autoantibodies (online supplemental tables S1 and S4). The brain damage markers neurofilament light polypeptide (NFL) and glial fibrillar acidic protein (GFAP) were extremely high in both CSF and plasma as symptoms peaked and gradually normalized during immunosuppression (online supplemental figure S3, tables S1 and S3). Tau and S-100B levels were moderately increased (online supplemental table S3). Collectively, these findings suggest severe axonal damage (NFL) and astrocyte injury (GFAP).^11^ Interferon-γ, tumor necrosis factor-α, and interleukin-6 levels were increased in CSF when symptoms peaked and normalized during recovery (online supplemental figure S3). The checkpoint proteins PD1, PD-L1, and Tim-3 showed a similar pattern (online supplemental figure S4). CTLA-4 and LAG-3 were not elevated.

### T cell characteristics

**Figure 2:**
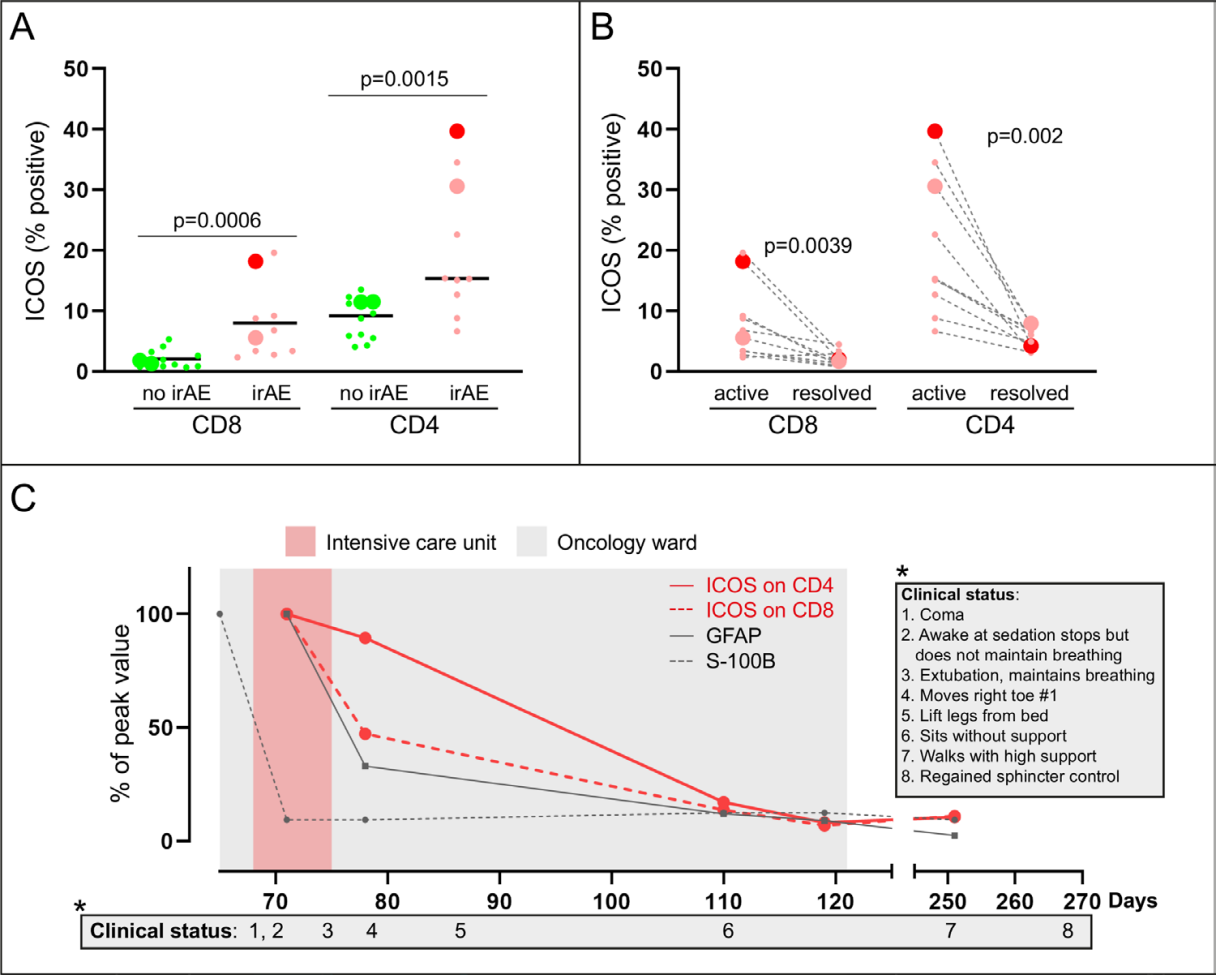
High proportion of ICOS-expressing CD4+ and CD8+T cells during active encephalomyelitis. Panel (A) shows higher proportion of T cells expressing the costimulatory receptor ICOS at the peak of symptoms in checkpoint inhibitor-treated patients with immune-related adverse events (irAE; pink dots and the big red dot which indicates the encephalitis patient) than in patients without irAE (no irAE; green dots) (large dots—double inhibition; small dots—single PD-1-inhibition) (Mann-Whitney U test). The encephalitis patient had the highest proportion of ICOS positive CD4+T cells and the second highest proportion of ICOS-expression on CD8+ cells. (B) shows that immunosuppression decreased the proportion of ICOS expressing CD8+ and CD4+T cells in the encephalitis patient as well as in patients with other irAE (Wilcoxon matched-pairs signed rank test). (C) shows that the proportion of ICOS expressing CD8 (dotted red line) and CD4 (solid red line) T cells decreased in parallel with clinical improvement (box) and with decrease in brain damage marker GFAP in blood. GFAP, glial fibrillar acidic protein; ICOS, inducible T cell costimulatory receptor; PD-1, programmed cell death 1.

**Figure 3:**
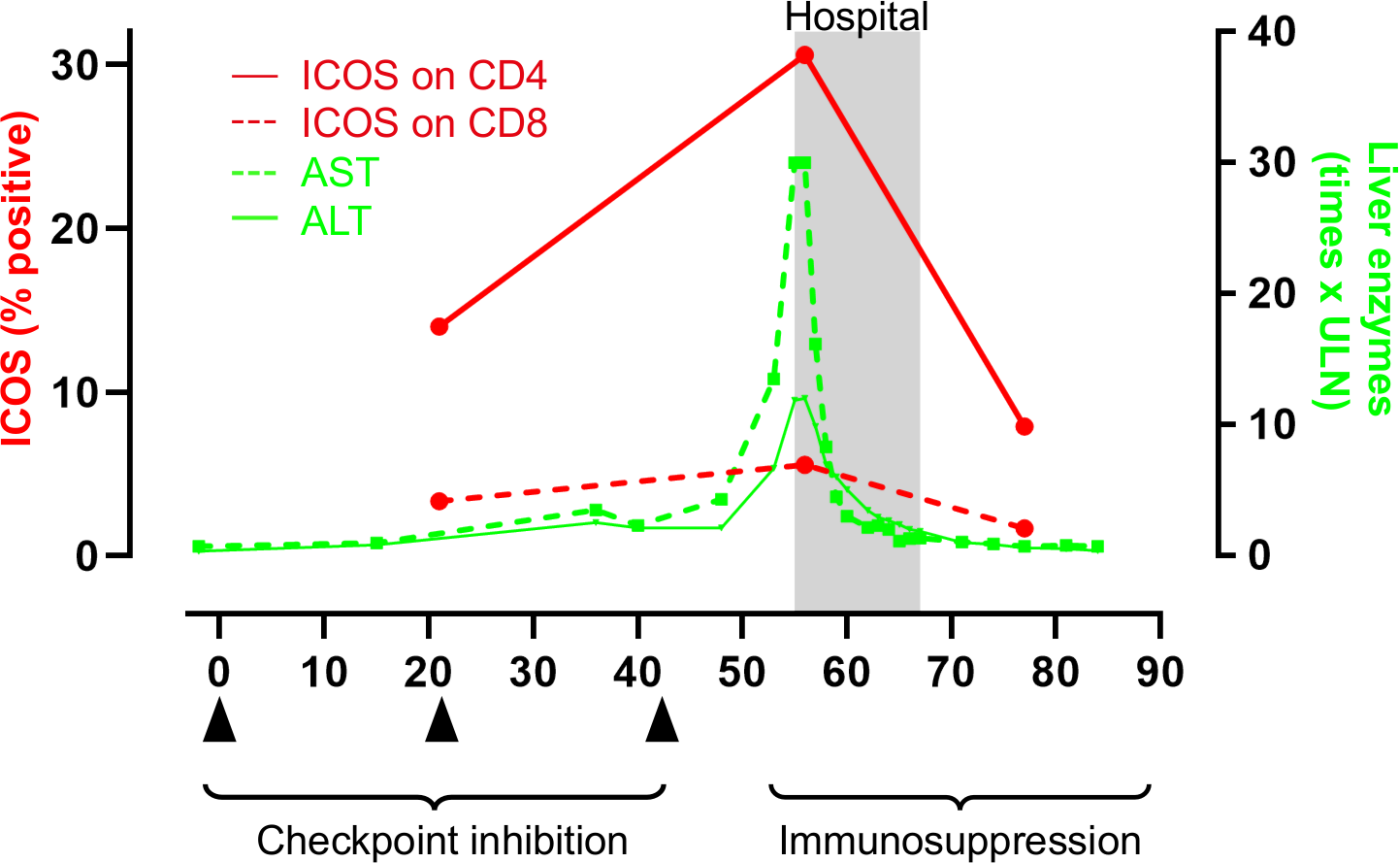
High proportion of ICOS-expressing CD4+T cells during severe checkpoint inhibitor-induced hepatitis The figure shows covariation of liver enzymes—aspartate transaminase (AST; solid green line) and alanine aminotransferase (ALT; dotted green line)—and ICOS expression on CD4+T cells (solid red line) before, at the peak of, and after severe checkpoint inhibitor–induced hepatitis (grade 4). ICOS on CD8+T cells (dotted red line) showed a similar, but less pronounced, covariation with liver enzymes. The black triangles indicate time points for double checkpoint inhibition with ipilimumab and nivolumab. ICOS, inducible T cell costimulatory receptor.

Flow cytometry of peripheral T cells was done on ICU day 2 and repeated four times. The analysis included T cell subtypes, activation markers, costimulatory receptors, inhibitory immune checkpoints, transcription factors, and attack enzymes (online supplemental table S2). In addition to our encephalomyelitis patient, T cell phenotype was also analyzed in patients with other irAE as well as in patients without irAE (table 1). The most striking observation in the encephalomyelitis patient was high expression of inducible T cell costimulatory receptor (ICOS) on all subtypes of CD4 +T helper cells (figure 2A) (online supplemental figure S5) and on CD8+ cytotoxic T cells. ICOS expression on T cells gradually normalized during immunosuppression and in parallel with clinical improvement (figure 2B–D) (online supplemental figure S5B, C). Similarly, high ICOS on CD4+ and CD8+T cells was detected also in patients with other irAE (figure 2A) and decreased when irAEs had resolved (figure 2B). One patient developed grade 4 hepatitis during treatment with double checkpoint inhibition. In this patient, one sample was analyzed before irAE, when liver enzymes were normal. Interestingly ICOS on CD4+ and CD8+T cells increased in parallel with liver enzymes and decreased again after immunosuppression, and subsequent normalization of liver enzymes (figure 3). Collectively, our data indicate that ICOS may promote development of irAE. High ICOS expression could not be explained by longer duration of checkpoint inhibition because the difference was significant also if patients with late irAE (>6 months, 4 patients) were excluded from the analysis (irAE vs non-irAE; ICOS on CD8+ T cells, p<0.010; ICOS on CD4 +T cells, p<0.027). In addition, the difference in ICOS expression was significant also when only patients treated with single PD-1 inhibition was compared (irAE vs non-irAE; ICOS on CD8 +T cells, p<0.0085; ICOS on CD4+T cells, p<0.0062). This indicates that the difference in ICOS was not specific for anti-CTLA-4 treatment. Full data on T cell characteristics is shown in online supplemental figure S6. In addition to ICOS, TIM-3 (an inhibitory checkpoint protein and activation marker) was significantly higher on CD8 +T cells in patients with irAE and normalized when irAE were resolved. Also, PD-1 expression on CD4+ T cells was significantly higher in patients with irAE. The patients with irAE had similar proportion of immunosuppressive regulatory T cells as checkpoint inhibitor-treated controls (online supplemental figure S7). There was no difference in total number of CD8+ cytotoxic T cells, CD4+T helper cells, B cells, or natural killer cells, between our patient and checkpoint inhibitor-treated controls (online supplemental figure S8).

## Discussion and conclusion

In this study, we demonstrate a specific T cell phenotype in a patient with encephalomyelitis as well as in patients with other severe irAE. The most striking feature is high expression of costimulatory receptor ICOS on CD4+ and CD8+T cells. In addition, our study shows that brain damage markers in blood can help in early diagnosis of encephalitis.

irAE are a diverse set of checkpoint inhibitor-induced autoimmune reactions but little is known about the mechanisms promoting irAE.^5^ Here, we identify high ICOS expression, on both CD4+ and CD8+ cells, during encephalomyelitis and other serious irAE. ICOS decreased when the irAE resolved suggesting that ICOS may promote irAE. In agreement, ICOS has been linked to the development of different autoimmune diseases.^12^ The association between T cell expression of ICOS and the clinical course of irAE is clear but it is important to clarify if the ICOS molecule promotes irAE. If this is the case, targeting ICOS with antagonists may constitute a therapeutic approach to dampen severe irAE; such as the near-fatal encephalomyelitis described here. However, it is possible that such an intervention, as well as the immunosuppressive treatments used in our patients, may increase the risk of tumor progression or recurrence because ICOS has also been identified as a mediator of response.^13 14^

Double immune checkpoint blockade is often more effective than PD-1 inhibition alone, as targeting CTLA-4 also activates CD4 +T cells.^15^ In mice, the absence of CTLA-4 promotes the expansion of ICOS-positive CD4+ effector T cells, which are important in mediating the response to CTLA-4 inhibition.^14–17^ Interestingly, high ICOS expression on CD4 +cells also promotes the development of neuromyelitis optica spectrum disorder,^18^ an autoimmune demyelinating disease of the central nervous system. The high levels of ICOS expression on CD4+ effector cells in our patient could help explain both the efficient eradication of tumor cells and the collateral damage to normal brain cells. Consistent with our data, activated CD4+ memory cells accumulated in inflamed brain tissue from a patient who died from checkpoint inhibitor-induced encephalitis.^7^ At autopsy, no signs of remaining melanoma brain metastases were found. In combination with previous clinical and experimental data, the findings in our case support a role for ICOS expression on CD4+ cells in mediating an aggressive immune reaction.

The current case shows that brain damage biomarkers in blood can help to diagnose encephalitis. Our patient had increased levels of the brain damage marker S-100B and CRP after two treatments, when he was asymptomatic and MRI showed no signs of encephalitis. S-100B and CRP peaked after the fourth and final treatment, when his encephalitis rapidly progressed. S-100B was analyzed because it is a melanoma marker. However, it was negative before treatment, and therefore the elevated level reflected treatment-induced brain damage and not progression of melanoma. Additional biomarkers were analyzed in the ICU and repeatedly during recovery. Most notably, the axonal damage marker NFL and the marker of astrocytic injury GFAP were extremely high in both blood and CSF and normalized during improvement. To facilitate diagnosis of encephalitis, we suggest that a set of brain damage markers in blood be included in laboratory panels taken during double-checkpoint inhibition. Our patient developed very severe encephalomyelitis and it needs to be investigated if brain damage markers in blood also indicate less severe cases of encephalitis.

Checkpoint inhibitor-induced encephalitis is a diagnostic challenge. Given our patient’s serious cancer diagnosis, oncologists and intensivists discussed whether he should be admitted to the ICU. The decision to do so was based on the argument that the clinical and radiologic findings were consistent with causes other than cancer progression, such as infection or neurotoxicity. At admission, the patient was unconscious and had central respiratory depression. He would have died without ICU treatment.

In conclusion, this study suggests a potential role for ICOS on CD4+ and CD8+T cells in mediating encephalitis and other serious irAE. In addition, our case suggests that brain damage markers in blood should be analyzed to facilitate early diagnosis of encephalitis.

## References

[1] Tawbi HA, Forsyth PA, Algazi A, et al. Combined nivolumab and ipilimumab in melanoma metastatic to the brain. N Engl J Med 2018;379:722–30. 10.1056/NEJMoa1805453 30134131 PMC8011001

[2] Hellmann MD, Paz-Ares L, Bernabe Caro R, et al. Nivolumab plus ipilimumab in advanced non-small-cell lung cancer. N Engl J Med 2019;381:2020–31. 10.1056/NEJMoa1910231 31562796

[3] Larkin J, Chiarion-Sileni V, Gonzalez R, et al. Five-year survival with combined nivolumab and ipilimumab in advanced melanoma. N Engl J Med 2019;381:1535–46. 10.1056/NEJMoa1910836 31562797

[4] Motzer RJ, Rini BI, McDermott DF, et al. Nivolumab plus ipilimumab versus sunitinib in first-line treatment for advanced renal cell carcinoma: extended follow-up of efficacy and safety results from a randomised, controlled, phase 3 trial. Lancet Oncol 2019;20:1370–85. 10.1016/S1470-2045(19)30413-9 31427204 PMC7497870

[5] Postow MA, Sidlow R, Hellmann MD. Immune-related adverse events associated with immune checkpoint blockade. N Engl J Med 2018;378:158–68. 10.1056/NEJMra1703481 29320654

[6] Dubey D, David WS, Reynolds KL, et al. Severe neurological toxicity of immune checkpoint inhibitors: growing spectrum. Ann Neurol 2020;87:659–69. 10.1002/ana.25708 32086972

[7] Johnson DB, McDonnell WJ, Gonzalez-Ericsson PI, et al. A case report of clonal EBV-like memory CD4^+^ T cell activation in fatal checkpoint inhibitor-induced encephalitis. Nat Med 2019;25:1243–50. 10.1038/s41591-019-0523-2 31332390 PMC6689251

[8] Wang DY, Salem J-E, Cohen JV, et al. Fatal toxic effects associated with immune checkpoint inhibitors: a systematic review and meta-analysis. JAMA Oncol 2018;4:1721–8. 10.1001/jamaoncol.2018.3923 30242316 PMC6440712

[9] Pohl D, Alper G, Van Haren K, et al. Acute disseminated encephalomyelitis: updates on an inflammatory CNS syndrome. Neurology 2016;87:S38–45. 10.1212/WNL.0000000000002825 27572859

[10] Broen JCA, van Laar JM. Mycophenolate mofetil, azathioprine and tacrolimus: mechanisms in rheumatology. Nat Rev Rheumatol 2020;16:167–78. 10.1038/s41584-020-0374-8 32055040

[11] Khalil M, Teunissen CE, Otto M, et al. Neurofilaments as biomarkers in neurological disorders. Nat Rev Neurol 2018;14:577–89. 10.1038/s41582-018-0058-z 30171200

[12] Edner NM, Carlesso G, Rush JS, et al. Targeting co-stimulatory molecules in autoimmune disease. Nat Rev Drug Discov 2020;19:860–83. 10.1038/s41573-020-0081-9 32939077

[13] Fu T, He Q, Sharma P. The ICOS/ICOSL pathway is required for optimal antitumor responses mediated by anti-CTLA-4 therapy. Cancer Res 2011;71:5445–54. 10.1158/0008-5472.CAN-11-1138 21708958

[14] Shi LZ, Goswami S, Fu T, et al. Blockade of CTLA-4 and PD-1 enhances adoptive T-cell therapy efficacy in an ICOS-Mediated manner. Cancer Immunol Res 2019;7:1803–12. 10.1158/2326-6066.CIR-18-0873 31466995

[15] Sharma P, Allison JP. Dissecting the mechanisms of immune checkpoint therapy. Nat Rev Immunol 2020;20:75–6. 10.1038/s41577-020-0275-8 31925406

[16] Wei SC, Levine JH, Cogdill AP, et al. Distinct cellular mechanisms underlie anti-CTLA-4 and anti-PD-1 checkpoint blockade. Cell 2017;170:1120–33. e1117. 10.1016/j.cell.2017.07.024 28803728 PMC5591072

[17] Wei SC, Sharma R, Anang N-AAS, et al. Negative co-stimulation constrains T cell differentiation by imposing boundaries on possible cell states. Immunity 2019;50:1084–98. 10.1016/j.immuni.2019.03.004 30926234 PMC6664799

[18] Xue Q, Li X, Gu Y, et al. Unbalanced expression of ICOS and PD-1 in patients with neuromyelitis optica spectrum disorder. Sci Rep 2019;9:14130. 10.1038/s41598-019-50479-4 31575949 PMC6773714

