# Supplementary material for: Early rise in brain damage markers and high ICOS expression in CD4+ and CD8+ T cells during checkpoint inhibitor-induced encephalomyelitis
Source: J Immunother Cancer. 2021 Jul 2;9(7):e002732. doi: 10.1136/jitc-2021-002732 (PMC8256743; doi:10.1136/jitc-2021-002732)
Supplement: Supplementary data [file jitc-2021-002732supp001.pdf]

SUPPLEMENTARY APPENDIX

CONTENTS

***SUPPLEMENTARY METHODS* 2**

***SUPPLEMENTARY FIGURES*.. 3**

Figure S1. Gating strategy for CD8+ and CD4+ lymphocytes and their sub-populations in blood ……3

Figure S2. Results of Search of the WHO Global Database VigiBase for Reported Cases of Encephalitis during Single- or Double-Checkpoint Inhibition …...4

Figure S3. Brain damage markers and cytokines in cerebrospinal fluid during active encephalomyelitis…………………………………………………………………………………..5

Figure s4. High levels of soluble checkpoint proteins PD-1, PD-L1 and Tim-3 in cerebrospinal fluid during active encephalomyelitis……………………………………………………………………………...........................6

Figure S5. High Proportion of ICOS-Expressing Th1, Th2, Th17, and Th1Th17 cells during active encephalomyelitis.. 7

Figure S6. T-cell characteristics during active encephalomyelitis and other irAEs..............................8

Figure S7. Proportion of regulatory T Cells during Active Encephalomyelitis and other irAEs............9

Figure S8. Total Number of Immune Cells during Active Encephalomyelitis and other irAEs 10

SUPPLEMENTARY TABLES* *11

Table S1. Laboratory Analyses of cerebrospinal fluid during intensive care and recovery.................11

Table S2. Antibodies used for flow cytometry.. 12

Table S3. Brain damage markers in blood during intensive care and recovery……………………........ 13

Table S4. No autoantibodies in blood or cerebrospinal fluid during active encephalomyelitis.........14

METHODS

Ethical considerations – The encephalitis patient and control patients received oral and written information about the purpose of blood samples and cerebrospinal fluid taken for research. All patients signed an informed consent form. The first research samples from the encephalitis patient was obtained when he was deeply unconscious in a respirator. The sample was taken after discussion with the regional ethics committee. Following their instruction, the patient was informed after recovery and at that point he approved analysis of samples for research purposes. If he had declined, the samples would have been destroyed. The research protocols were approved by the Regional Ethics Review Board in Gothenburg (numbers 151-16 and 433-11). The encephalitis patient gave an additional separate consent to publish this case.

Flow cytometry – Peripheral blood mononuclear cells (PBMCs) were separated from heparinized whole blood, stained with fluorochrome-conjugated antibodies (Table S2) and analyzed in a FACSLyric flow cytometer. CD4 and CD8 T-cell subsets were defined by gating with FlowJo software. Total counts of T-cells, B-cells, and natural killer cells (NK cells) were determined using TruCount. PBMCs from checkpoint inhibitor treated melanoma patients with (n=9) or without (n=12) immune related adverse events were used as controls. Checkpoint proteins in CSF were analyzed with LEGENDplex bead-based multiplex assay panels; data was acquired on a FACSVerse flow cytometer, and concentrations were calculated with FCAP Array software. CSF from patients with systemic lupus erythematosus without autoimmune manifestations in the brain were used as controls (n=4).

Brain damage markers – The S-100B concentrations in CSF and serum were measured by immunoassay on the cobas Elecsys platform. CSF concentrations of neurofilament light polypeptide (NFL) and glial fibrillary acidic protein (GFAP) were measured with in-house enzyme-linked immunosorbent assays as described.[1,2] CSF tau concentration was measured with a Lumipulse immunoassay. Plasma concentrations of NFL, GFAP, and tau were measured with ultrasensitive single-molecule array technology and commercially available kits. CSF and serum concentrations of albumin and IgG were measured by nephelometry on the cobas Elecsys platform. Oligoclonal IgG bands in serum and CSF were visualized by isoelectric focusing in a polyacrylamide gel and silver staining as described.[3]

References

1. Gaetani L, Hoglund K, Parnetti L, et al. A new enzyme-linked immunosorbent assay for neurofilament light in cerebrospinal fluid: analytical validation and clinical evaluation. Alzheimers Res Ther 2018;10:8.
2. Rosengren LE, Wikkelso C, Hagberg L. A sensitive ELISA for glial fibrillary acidic protein: application in CSF of adults. J Neurosci Methods 1994;51:197-204.
3. Blennow K, Wallin A, Davidsson P, et al. Intra-blood-brain-barrier synthesis of immunoglobulins in patients with dementia of the Alzheimer type. Alzheimer Dis Assoc Disord 1990;4:79-86

Bjursten S, *et al. J Immunother Cancer* 2021; 9:e002732. doi: 10.1136/jitc-2021-002732

SUPPLEMENTARY FIGURES

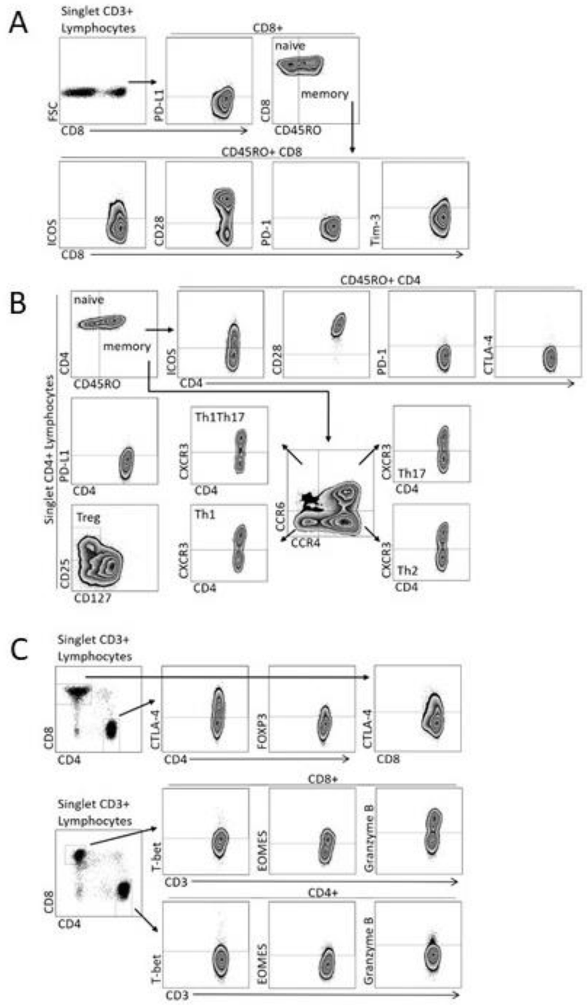

Figure S1. Gating strategy for CD8+ and CD4+ lymphocytes and their sub-populations in blood

Panel A. Singlet CD3+ lymphocytes were gated for CD8+ T cells and then gated for PD-L1+ cells. CD8+ T cells were divided into CD45RO- (naïve) and CD45RO+ (memory) cells, and memory cells were gated according to the expression of ICOS, CD28, PD-1 and Tim-3. To set the gates for the CD8+ T-cell subtypes we used Fluorescence Minus One (FMO) except for PD-L1, where an isotype control was used.Panel B. Singlet CD4+ lymphocytes were gated for PD-L1 and for CD25 and CD127. CD4+ T cells were divided into CD45RO- (naïve) and CD45RO+ (memory) cells, and memory cells were gated according to the expression of ICOS, CD28, PD-1 and CTLA-4. Memory cells were also gated into Th1, Th2, Th17 and Th1Th17 based on the expression of CCR4, CCR6 and CXCR3. To set the gates for the CD8+ T-cell subtypes we used FMO except for PD-L1, where an isotype control was used. Panel C. Singlet CD3+ were first gated into CD8+ and CD4+ T cells, followed by gating for the intracellular molecules CTLA-4, FOXP3, T-bet, EOMES and Granzyme B. Isotype controls were used for T-bet, EOMES and Granzyme B, whereas FMO was used for CTLA-4. The cut off for FOXP3 positivity in CD4+ cells was determined based on FOXP3 expression in CD25neg gated CD4+ cells.

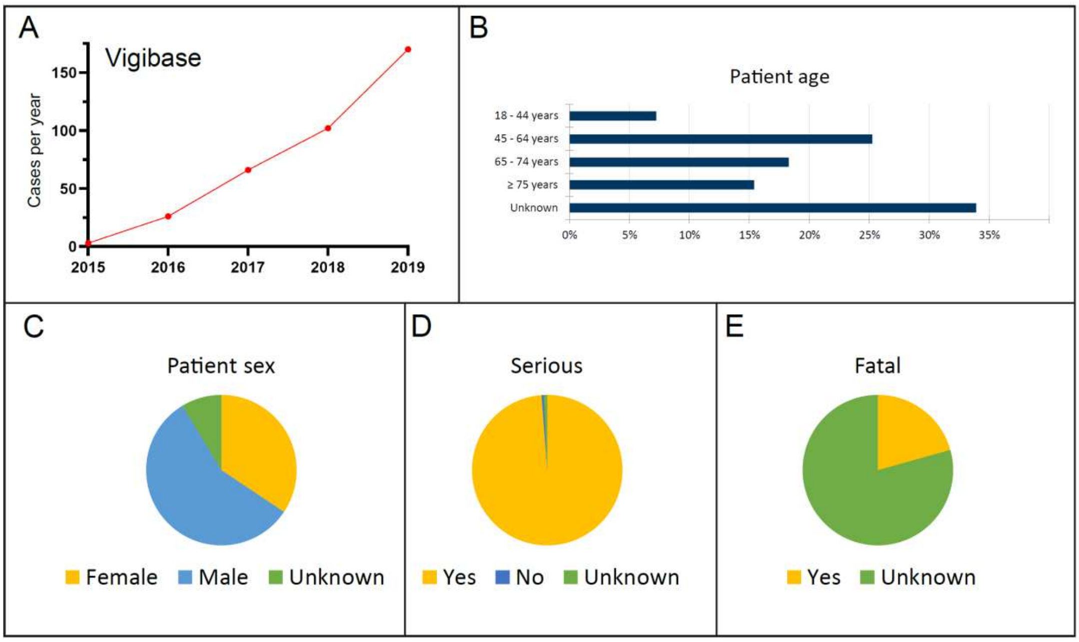

Figure S2. Results of search of the WHO Global Database VigiBase for reported cases of encephalitis during single- or double-checkpoint Inhibition.

Panel A shows annual number of reported cases from 2015 through 2019. Panel B to D shows the ages of the cases reported in panel (B), the sex of the patients (C), the severity of their condition (D) and the outcome (E).

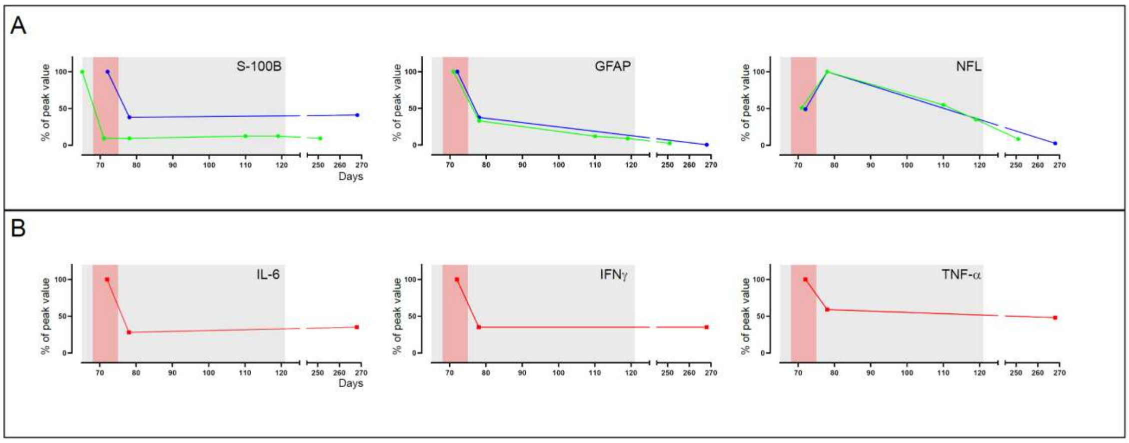

Figure S3. Brain damage markers and cytokines in cerebrospinal fluid during active encephalomyelitis

Panel A shows relative concentrations (% of highest value) of brain damage markers in blood (green line) and cerebrospinal fluid (CSF) (blue line) during encephalomyelitis and recovery. Panel B shows relative concentrations of cytokines in CSF.

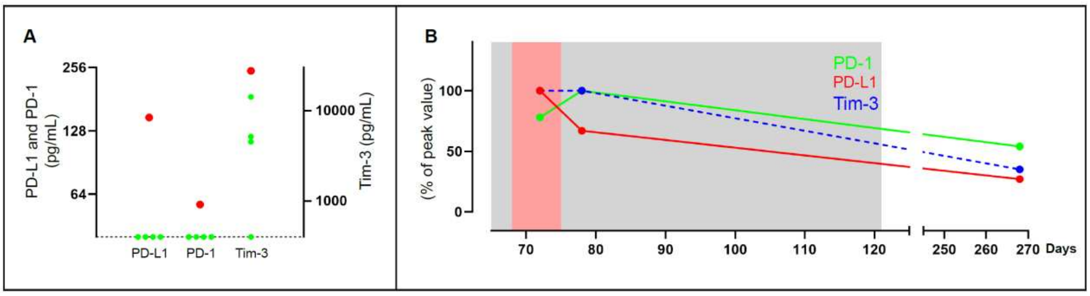

Figure S4. High levels of soluble checkpoint proteins PD-1, PD-L1 and Tim-3 in cerebrospinal fluid during active encephalomyelitis

Panel A shows cerebrospinal fluid (CSF) concentrations of the soluble checkpoint proteins PD-1, PD-L1, and Tim-3 at the peak of symptoms in the encephalomyelitis patient (big red dot) than in CSF from patients with autoimmune systemic lupus erythematosus without encephalitis (green dots). The base line on the y-axes indicates detection level (PD-1 and PD-L1 – 40 pg/mL; Tim-3 – 400 pg/mL). Panel B shows that immunosuppression decreased PD-1, PD-L1 and Tim-3 concentrations in CSF in the encephalitis patient.

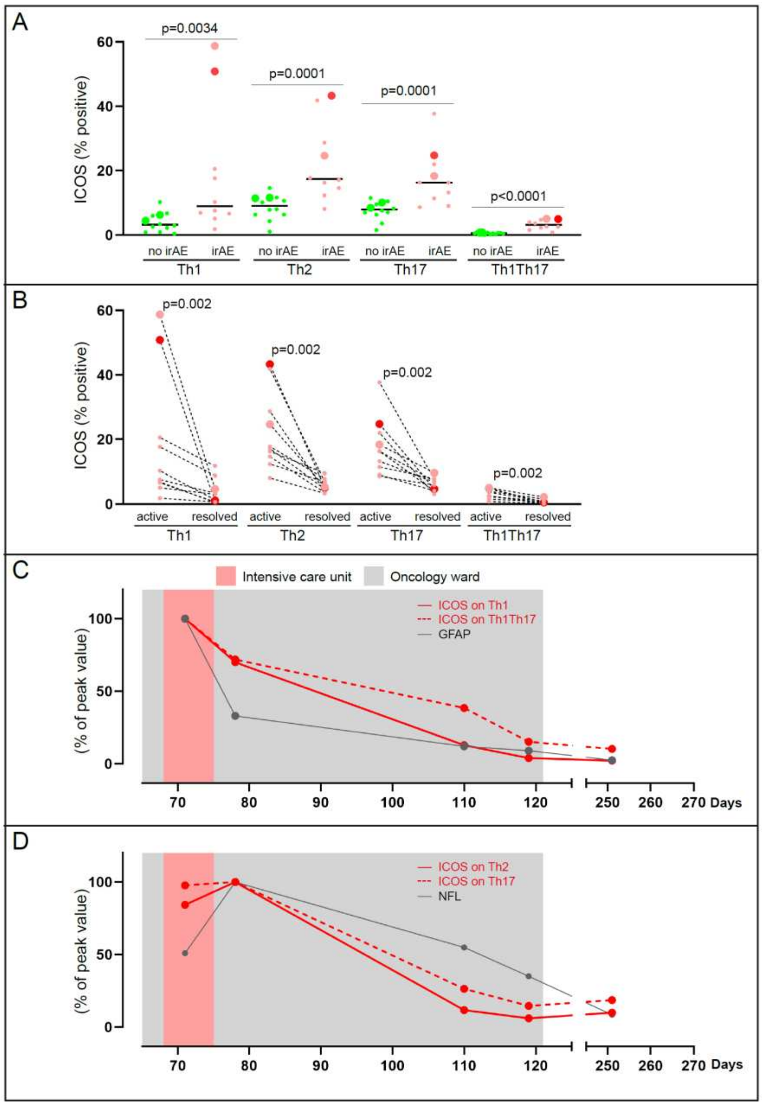

Figure S5. High proportion of ICOS-expressing Th1, Th2, Th17, and Th1Th17 cells during active encephalomyelitis.

Panel A shows higher proportion of CD4+ cell subtypes (Th1, Th2, Th17, and Th1Th17) that express the co-stimulatory receptor ICOS at the peak of symptoms in the encephalomyelitis patient (big red dot) than in checkpoint inhibitor-treated patients with other immune related adverse events (pink dots) or in patients without immune-related adverse events (green dots) (Mann Whitney test). Big dots indicate double inhibition and small dots single inhibition. Panel B shows that immunosuppression decreased the proportion of CD4+ T cell subtypes that express ICOS in the encephalitis patient as well as in patients with other immune-related adverse events (Wilcoxon matched-pairs signed rank test). Panel C shows that the proportion of ICOS-expressing Th1 and Th1Th17 cells decreased in parallel with clinical improvement and with decrease in glial injury marker GFAP in blood. Panel D shows that the proportion of ICOS-expressing Th2 and Th17 cells increased initially during immunosuppression and then decreased. A similar dynamic was seen in axonal damage marker NFL in blood.

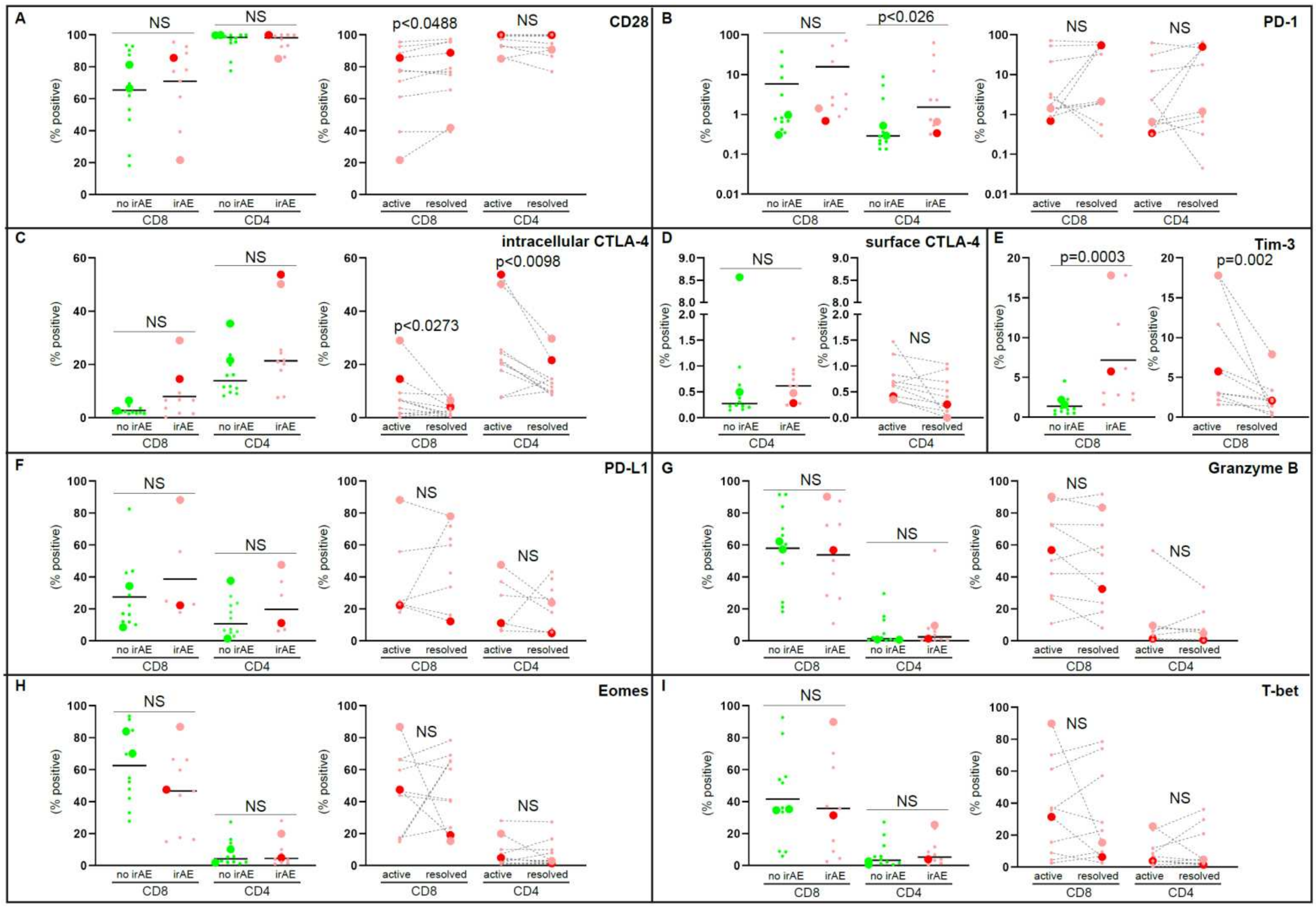

Figure S6. T cell characteristics during active encephalomyelitis and other irAEs

Panels A-I shows T cell characteristics at the peak of symptoms in the encephalitis patient (big red dot), patients with other immune related adverse events (irAE; pink dots) and patients without irAE (no irAE; green dots). Big dots indicate double inhibition and small dots single inhibition. Also shown is the effect of immunosuppression on proportion of T-cell subtypes in patients with irAE (active vs resolved). Proportion of CD8+ and CD4+ T cells that express co-stimulatory checkpoint protein CD28 (A); co-inhibitory checkpoints PD-1 (B), intracellular (C) and cell-surface CTLA-4 (D), and Tim-3 (E); PD-1 ligand PD-L1 (F); attack enzyme Granzyme B (G); activating transcription factors T-bet (H) and Eomes (I) are shown. The proportion of CD8+ and CD4+ T cells that express intracellular CTLA-4 decreased after immunosuppression (C). A higher proportion of CD8 cells expressed Tim-3 in patients with immune related adverse events and Tim-3 expression decreased after immunosuppression (E). P values; Mann Whitney test (no irAE vs. irAE) or Wilcoxon matched-pairs signed rank test (active vs. resolved); NS – non significant.

Bjursten S, *et al. J Immunother Cancer* 2021; 9:e002732. doi: 10.1136/jitc-2021-002732

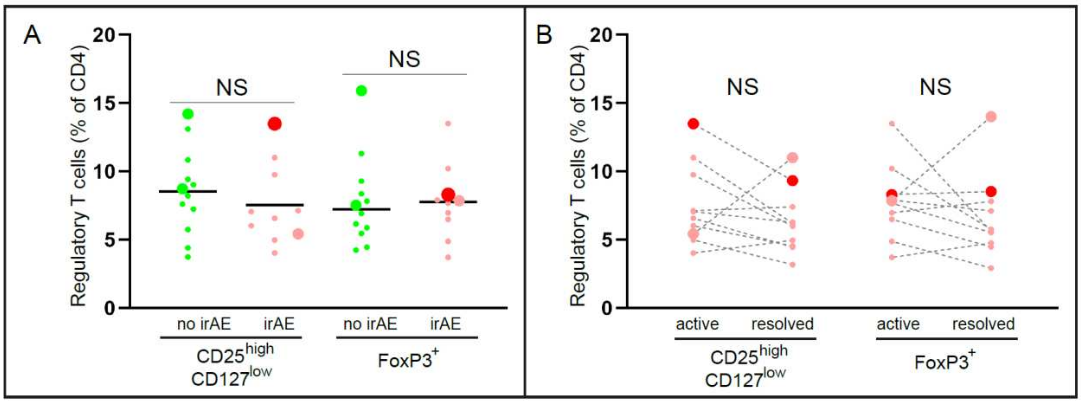

Figure S7. Proportion of regulatory T cells during active encephalomyelitis and other irAEs

Panel A shows no difference in two populations of regulatory T cells, $CD4^{+}CD25^{high}CD127^{low}$ and $CD4^{+}FoxP3^{+}$, in patients with (pink dots) or without immune related adverse events (green dots). Big dots indicate double inhibition and small dots single inhibition. The encephalomyelitis patient is indicated by a big red dot. Panel B shows no difference in $CD4^{+}FoxP3^{+}$ cells or in $CD4^{+}CD25^{high}CD127^{low}$ regulatory T cells after immunosuppression (Wilcoxon matched-pairs signed rank test).NS – non significant.

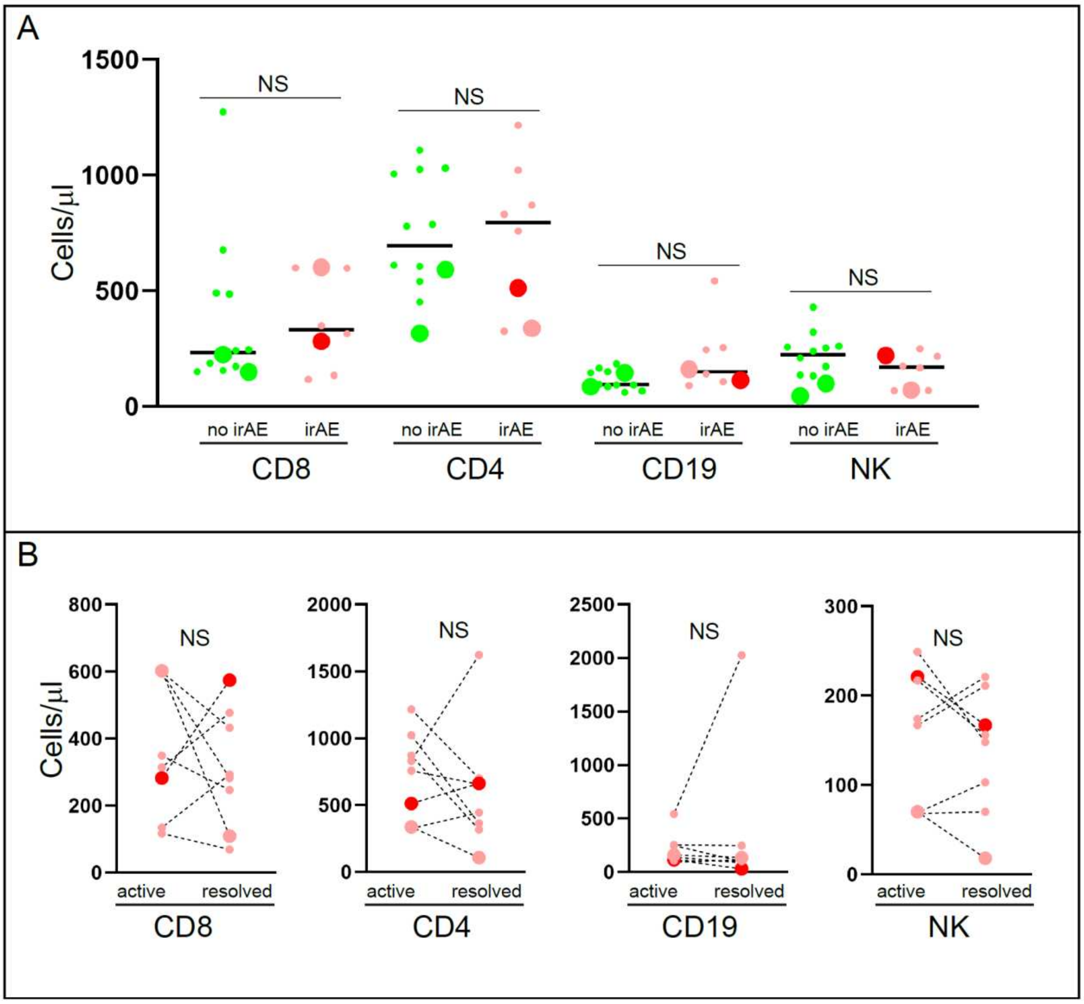

Figure S8. Total number of immune cells during active encephalomyelitis and other irAEs

Panel A shows no difference in total numbers of CD8 cytotoxic T cells, CD4 T helper cells, B cells, or natural killer cells (NK) in patients with (pink dots) or without immune related adverse events (green dots)(Mann Whitney test).c Big dots indicate double inhibition and small dots single inhibition. The encephalomyelitis patient is indicated by a big red dot. Panel B shows no change in any of these cell populations after immunosuppression (Wilcoxon matched-pairs signed rank test). NS – non significant.

SUPPLEMENTARY TABLES

Table 1. Laboratory analyses of cerebrospinal fluid during intensive care and recovery

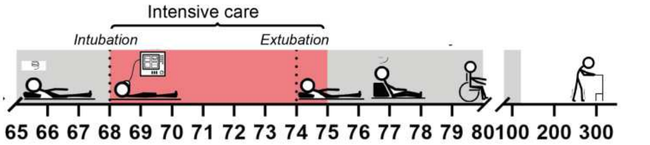

**Laboratory test** (reference values)	**Day 68**	**Day 72**	**Day 75**	**Day 268**
GFAP (<1250 ng/L)		92800	30400	490
NFL (<1850 ng/L)		85400	174000	4580
Tau protein (<400 ng/L)		484	1730	240
Albumin (<420 mg/L)	2880	814.8	505.8	170.6
Qalb (<10.2 x 10^{-3})	106.8 x 10^{-3}	29.1 x 10^{-3}	18.7 x 10^{-3}	5.9 x 10^{-3}
S-100B(<1,7 µg/L)		2.79	1.07	1.13
IgG (<60 mg/L)		359.6	255.2	34.6
IEF (bands in CSF only)		4 - 5	4 - 5	8 - 10
VZV	Negative			
HSV type 1	Negative			
HSV type 2	Negative			
Enterovirus	Negative			
CNS-infection, 14 agents*	Negative			
Bacterial culture	Negative			
16S rRNA gene	Negative			
Glucose (<2/3 of blood glc)L	Normal			
Lactate (0.5 - 1.8 mM/L)	5.1			
Lymphocytes (<4 x 10^6/L)	16		11	15
Monocytes(<3 x 10^6/L)	<3		<3	<3
Neutrophils (<3 x 10^6/L)	<3		<3	<3
Erythrocytes (<5 x 10^6/L)	24		42	<5
IL-1β (<5 pg/mL)		<5	5.4	8.2
IL-2 (<16 pg/mL)		<16	<16	<16
IL-4 (<16 pg/mL)		<0.25	<0.25	<0.25
IL-5 (<8 pg/mL)		3.9	<3.9	<3.9
IL-6 (<50 pg/mL)		12	3.4	4.2
IL-8 (<90 pg/mL)		360	190	150
IL-10 (<20 pg/mL)		12	9	5.9
IL-12 (<7.8 pg/mL)		<7.8	<7.8	<7.8
IFN-γ (<7.8 pg/mL)		22	<7.8	<7.8
GM-CSF (<7.8 pg/mL)		<7.8	<7.8	<7.8
TNF-α (<25 pg/mL)		17	10	8.2
IL-18 (140-500 pg/mL)			65	

GFAP, Glial fibrillary acidic protein; NFL, Neurofilament light polypeptide; Qalb, albumin quotient (CSF/serum); IEF, Isoelectric Focusing; VZV, Varicella zoster virus; HSV, Herpes simplex virus ; rRNA, Ribosomal ribonucleic acid; IL, Interleukin; IFN-γ, Interferon gamma; GM-CSF, Granulocyte-macrophage colony-stimulating factor; TNF-α, Tumor necrosis factor alpha.

*Filmarray meningitis panel: Herpes simplex type 1 and 2, Varicella zooster virus, Enterovirus, Human herpes virus 6, Human parechovirus, Cytomegalovirus, Streptococcus pneumonia, Hemophilus

Table S2. Antibodies used for flow cytometry

**antigen**	**fluorochrome**	**isotype**	**Manufacturer**	**clone**
CD4	APC-H7	IgG1	BD	SK3
CD25	BV421	IgG1	Biolegend	BC96
CD25	APC	IgG1	BD	2A3
CD127	PerCP-Cy5.5	IgG1	BD	HIL-7R-M21
CD45RO	FITC	IgG2a	Biolegend	UCHL1
PD-1	PE	IgG1	Biolegend	EH12.2H7
Foxp3	PE	IgG2a	eBioscience	PCH101
CTLA-4	biotin	IgG2a	BD	BNI3
CCR4	PE-Cy7	IgG1	Biolegend	L291H4
CCR6	APC	IgG2b	Biolegend	G034E3
CXCR3	BV421	IgG1	Biolegend	G025H7
CD8	PE-Cy7	IgG1	BD	RPA-T8
CD3	APC-H7	IgG1	BD	SK7
CD4	BV421	IgG1	BD	SK3
CD3	BV421	IgG1	BD	UCHT1
CD28	APC	IgG1	Biolegend	CD28.2
TIM-3	BV421	IgG1	Biolegend	F38-2E2
ICOS	PerCP-eFluor710	IgG1	eBioscience	ISA-3
T-bet	PE	IgG1	Biolegend	4B10
Granzyme B	PerCP-Cy5.5	IgG1	Biolegend	QA16A02
EOMES	eFluor660	IgG1	eBioscience	WD1928
PD-L1	APC	IgG2b	Biolegend	29E.2A3
isotype	APC	IgG2b	Biolegend	MPC-11
isotype	PerCP-Cy5.5	IgG1	Biolegend	MOPC-21
isotype	PE	IgG1	Biolegend	MOPC-21
isotype	eFluor660	IgG1	eBioscience	

Bjursten S, *et al. J Immunother Cancer* 2021; 9:e002732. doi: 10.1136/jitc-2021-002732

Table S3. Brain damage markers in blood during intensive care and recovery

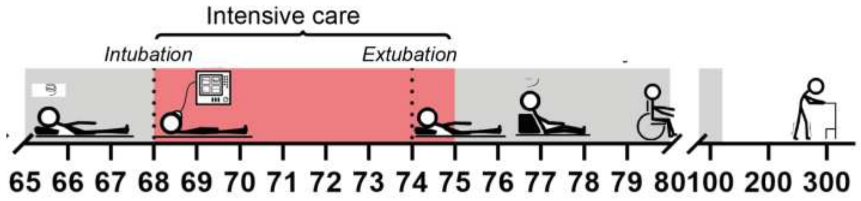

**Laboratory test (reference value)**	**Day 65**	**Day 70**	**Day 71**	**Day 78**	**Day 110**	**Day 119**	**Day 251**
**GFAP (<210 ng/L)**			12208	1091.8	405.9	291	80
**NFL (< 30 ng/L)**			153.7	301.7	165.7	105.7	25.7
**Tau (< 5 ng/L)**			3.1	2.6	3.7	3.2	3.3
**S-100B(<0.1 μg/L)**	0.31	0.06	0.03	0.02	0.04	0.04	

GFAP, Glial fibrillary acidic protein; NFL, Neurofilament light polypeptide.

Table S4. No autoantibodies in blood or cerebrospinal fluid during active encephalomyelitis

**Antinuclear antibody screen (blood)**	**Result**
ANA	Negative
Anti-SS-A60	Negative
Anti-SS-A52	Negative
Anti-SS-B	Negative
Anti-Sm	Negative
Anti-RNP	Negative
Anti-Scl-70	Negative
Anti-Jo-1	Negative
Anti-dsDNA	Negative
Anti Cent B	Negative
Anti-Ribo P	Negative
	Negative
**Antibodies associated with autoimmune encephalitis (cerebrospinal fluid)**	
Anti-NMDA-receptor	Negative
Anti-LGI1	Negative
Anti-CASPR2	Negative
Anti-GABA-B	Negative
Anti-VGCC PQ-typ	Negative
Anti-AMPA 1/2-receptor	Negative
Anti-DPPX	Negative
**Antibodies against neuronal antigens (cerebrospinal fluid)**	
Anti-CV2	Negative
Anti-Amphiphysin	Negative
Anti-Ma2/Ta	Negative
Anti-Tr	Negative
Anti-Recoverin	Negative
Anti SOX1	Negative
Anti-Zic4	Negative
Anti-Aquaporin-4 (NMO)	Negative
**Paramalignant antibodies (cerebrospinal fluid)**	
Anti-Hu Ri Yo	Negative
Anti-PCA2	Negative
